# Prognostic Significance of *POLE* Proofreading Mutations in Endometrial Cancer

**DOI:** 10.1093/jnci/dju402

**Published:** 2014-12-12

**Authors:** David N. Church, Ellen Stelloo, Remi A. Nout, Nadejda Valtcheva, Jeroen Depreeuw, Natalja ter Haar, Aurelia Noske, Frederic Amant, Ian P. M. Tomlinson, Peter J. Wild, Diether Lambrechts, Ina M. Jürgenliemk-Schulz, Jan J. Jobsen, Vincent T. H. B. M. Smit, Carien L. Creutzberg, Tjalling Bosse

**Affiliations:** **Affiliation of authors:**Molecular and Population Genetics Laboratory, Wellcome Trust Centre for Human Genetics, University of Oxford, Oxford, UK (DNC, IPMT); Oxford Cancer Centre, Churchill Hospital, Oxford, UK (DNC); Department of Pathology (ES, NtH, VTHBMS, TB) and Department of Clinical Oncology (RAN, CLC), Leiden University Medical Center, Leiden, the Netherlands; Institute of Surgical Pathology, University Hospital Zurich, Zurich, Switzerland (NV, AN, PJW); Vesalius Research Center (VRC), VIB, Leuven, Belgium (JD, DL); Laboratory of Translational Genetics, Department of Oncology, KU Leuven, Belgium (FA, DL); Department of Obstetrics & Gynecology, Division of Gynecologic Oncology, University Hospitals Gasthuisberg, Leuven, Belgium (FA); Genomic Medicine Theme, Oxford Comprehensive Biomedical Research Centre, Wellcome Trust Centre for Human Genetics, University of Oxford, Oxford, UK (IPMT); Department of Radiation Oncology, University Medical Centrum Utrecht, the Netherlands (IMJS); Department of Radiation Oncology, Medisch Spectrum Twente, Enschede, the Netherlands (JJJ).

## Abstract

**Background:**

Current risk stratification in endometrial cancer (EC) results in frequent over- and underuse of adjuvant therapy, and may be improved by novel biomarkers. We examined whether *POLE* proofreading mutations, recently reported in about 7% of ECs, predict prognosis.

**Methods:**

We performed targeted *POLE* sequencing in ECs from the PORTEC-1 and -2 trials (n = 788), and analyzed clinical outcome according to *POLE* status. We combined these results with those from three additional series (n = 628) by meta-analysis to generate multivariable-adjusted, pooled hazard ratios (HRs) for recurrence-free survival (RFS) and cancer-specific survival (CSS) of *POLE-*mutant ECs. All statistical tests were two-sided.

**Results:**

*POLE* mutations were detected in 48 of 788 (6.1%) ECs from PORTEC-1 and-2 and were associated with high tumor grade (*P* < .001). Women with *POLE*-mutant ECs had fewer recurrences (6.2% vs 14.1%) and EC deaths (2.3% vs 9.7%), though, in the total PORTEC cohort, differences in RFS and CSS were not statistically significant (multivariable-adjusted HR = 0.43, 95% CI = 0.13 to 1.37, *P* = .15; HR = 0.19, 95% CI = 0.03 to 1.44, *P* = .11 respectively). However, of 109 grade 3 tumors, 0 of 15 *POLE*-mutant ECs recurred, compared with 29 of 94 (30.9%) *POLE* wild-type cancers; reflected in statistically significantly greater RFS (multivariable-adjusted HR = 0.11, 95% CI = 0.001 to 0.84, *P* = .03). In the additional series, there were no EC-related events in any of 33 *POLE*-mutant ECs, resulting in a multivariable-adjusted, pooled HR of 0.33 for RFS (95% CI = 0.12 to 0.91, *P* = .03) and 0.26 for CSS (95% CI = 0.06 to 1.08, *P* = .06).

**Conclusion:**

*POLE* proofreading mutations predict favorable EC prognosis, independently of other clinicopathological variables, with the greatest effect seen in high-grade tumors. This novel biomarker may help to reduce overtreatment in EC.

Endometrial cancer (EC) is the commonest gynecological malignancy in the Western world, and is rising in incidence because of increasing obesity and ageing of the population (1). Most cases (80%) are detected at an early stage (FIGO stage I) (2) because of early symptoms. The standard management of EC consists of hysterectomy and bilateral salpingo-oophorectomy with or without postoperative vaginal brachytherapy (VBT) or pelvic external beam radiotherapy (EBRT) depending on recurrence risk (3,4). Women with grade 3 cancers or advanced disease are increasingly treated with adjuvant chemotherapy (5,6), the role of which will be further defined by ongoing studies. Current risk stratification is based on both clinical (age) and pathologic factors (FIGO stage, tumor type, grade, and lymphovascular space invasion [LVSI]). Despite refinement in the use of postoperative treatment in EC over the last two decades, over- and underusage of adjuvant therapy remains a clinical problem. Approximately seven patients with stage I EC with risk factors need to receive VBT to prevent one recurrence, while 8% to 10% patients develop distant metastases that may have been prevented with adjuvant chemotherapy (3–6). Consequently, the identification of molecular markers predictive of recurrence risk or treatment benefit beyond current clinicopathological factors would represent a major advance (7). While studies have investigated the prognostic significance of several molecular alterations involved in endometrial carcinogenesis, including microsatellite instability (MSI), *PIK3CA* and *TP53* mutation (8,9), to date none have been incorporated into routine clinical practice.

We recently showed that germline variants in the exonuclease domain of the DNA polymerases *POLE* and *POLD1* predispose to cancer, including EC, by impairing polymerase proofreading and greatly increasing the rate of base substitution mutations (10). We subsequently demonstrated that somatic *POLE* proofreading mutations are found in about 7% of sporadic ECs, where they strongly associate with high tumor grade (11). Similar findings have been reported in parallel by The Cancer Genome Atlas (TCGA), which also demonstrated that while *POLE* mutations were only found in endometrioid tumors, they were not inversely associated with *TP53* mutation (12). These pathogenic *POLE* proofreading mutations, about 90% of which cluster in exons 9 and 13, localize to amino acids within, or close to, conserved motifs essential for proofreading function (11,12). In keeping with this, *POLE* proofreading-mutant ECs are ultramutated, with a base substitution mutation frequency among the highest in human tumors (13).

Different forms of genomic instability in cancers are known to be associated with clinicopathological features, including prognosis (14–16). Favorable outcome of women with *POLE*-mutant ECs has been suggested (12), but only reached statistical significance when limited to analysis of grade 3 tumors in a recent report (17), and current evidence is insufficient to inform practice (18). In this study, we have analyzed associations between *POLE* proofreading mutations, recurrence-free and cancer-specific survival in two large, randomized controlled trials (PORTEC-1 and -2) (3,4) of early-stage (FIGO stage I), (high-) intermediate risk EC, with central pathology review and mature follow-up data, and in three additional smaller EC series (9,12,19).

## Methods

### PORTEC Study Details

Details of the PORTEC-1 and PORTEC-2 studies have been published previously (see the Supplementary Materials, available online) (3,4). PORTEC-1 compared pelvic EBRT with no additional treatment (NAT) in 715 women with intermediate risk, stage I EC recruited between June 1990 and December 1997 (3). PORTEC-2 randomly assigned 427 women with high-intermediate risk stage I/IIA EC between May 2002 and September 2006 to either EBRT or VBT following surgery (4). The median (range) duration of follow-up was 159.6 (33.6–222) months in PORTEC-1 and 89 (18–122) months in PORTEC-2. The PORTEC study protocols were approved by the Dutch Cancer Society and by the medical ethics committees at participating centers. All patients provided written informed consent to study participation and treatment.

### Additional EC Series

The Leuven (n = 187) (9), Zurich/Basel (n = 267) (19), and TCGA (n = 373) (12) series have also been reported previously (Supplementary Materials, available online). The Leuven and TCGA cohorts were collected prospectively, while Zurich/Basel cases were identified retrospectively. These sets included both endometrioid and nonendometrioid ECs (EECs and NEECs), and also included patients with stage III/IV disease (21.8–31.2%). Central pathology review was mandated in both TCGA and the Zurich/Basel cohorts, while the Leuven cases were reviewed by a single academic pathologist. Patients in the Leuven and Zurich/Basel cohorts were managed according to standard protocols, while treatment in the TCGA series was at the discretion of the attending physician: median (range) follow-up in each was 29 (1–184) months, 46 (1–173) months, and 28.7 (0.6–185.6) months, respectively. Follow-up data varied between series. RFS data were available for the Leuven and TCGA series, but not the Zurich/Basel set, while CSS data were available for the Leuven and the Zurich/Basel series, but absent from the TCGA study. Collection and analysis of the Leuven and Zurich/Basel series were approved by the scientific ethics committee from all centers (UZ Leuven Medical ethics committee and KEK-ZH-NR: 2010-0358, respectively). Ethical approval for anonymized tumor molecular analysis was granted by Oxfordshire Research Ethics Committee B (Approval No. 05\Q1605\66).

### Demographic and Clinicopathological Variables

Baseline demographic and clinicopathological variables were treated as either categorical (eg, grade, stage, EEC vs NEEC) or continuous (age) as appropriate. All analyses were based on data from central pathology review.

### Molecular Analysis

Tumor DNA was extracted from formalin-fixed, paraffin-embedded (FFPE) blocks from 434 (60.1%) and 398 (93.2%) ECs from the PORTEC-1 and -2 studies, respectively. There were no statistically significant differences between the biomarker and the total study populations in demographic/clinicopathological characteristics, treatment or survival. DNA was extracted from 187 fresh-frozen ECs in the Leuven set, 373 fresh-frozen ECs in the TCGA set, and 260 FFPE ECs in the Zurich/Basel set (Supplementary Methods, available online). The Leuven, Zurich/Basel, and TCGA biomarker cohorts were similar to the total series population in each case (Supplementary Methods, available online).

Sanger sequencing of *POLE* exons 9 and 13 (Supplementary Table 1, available online) was successful in over 94% of cases. For TCGA, we extracted *POLE* mutation data from publicly available whole-exome sequencing data (https://tcga-data.nci.nih.gov/tcga/) (12). We defined pathogenic *POLE* proofreading mutations as variants absent from public germline sequence databases (http://evsgs.washington.edu/EVS/; http://www.1000genomes.org/) and previously confirmed as somatic variants associated with tumor ultramutation (11,12,20,21), with the exception of one novel variant predicted to perturb protein function by SIFT (http://sift.jcvi.org) and Mutation Assessor (http://mutationassessor.org). All variants were confirmed in at least duplicate independent polymerase chain reaction and sequencing reactions.

### Statistical Analysis

Analyses performed and reported in this biomarker study are listed in Supplementary Table 2 (available online) in accordance with published guidelines (22,23). For analysis of the association of *POLE* mutation with outcome, our primary endpoint was recurrence-free survival (RFS), with secondary endpoints of cancer-specific survival (CSS), and overall survival (OS). In the PORTEC studies, RFS was defined as the time from random assignment to relapse, with censoring at last contact or death in case of no recurrence. CSS was measured as the time from random assignment to death from EC, with censoring at date of last contact or noncancer death. OS was measured as the time from random assignment to death from any cause, with censoring at date of last contact in patients still alive. The same criteria were used in the additional series, with the exception that survival measurements were from time of diagnosis. Survival curves were plotted using the Kaplan-Meier method and compared by the log-rank test. We used Cox proportional hazards models to calculate hazard ratios (HRs) for RFS, CSS, and OS of *POLE*-mutant ECs relative to *POLE* wild-type tumors by univariable analyses and, following adjustment for baseline characteristics and prognostic factors (age, tumor type, grade, LVSI, depth of myometrial invasion, and treatment), by multivariable analyses (see Supplementary Methods and Supplementary Tables 3–6, available online). Proportionality of hazards in Cox models was confirmed by visual inspection of complementary log plots or by interaction terms of covariables and (log)time. In view of the similar patient populations, the limited number of cancer-related events in both PORTEC trials, and the modest frequency of *POLE* mutations, the PORTEC studies were combined for most analyses. For Cox regression analysis of the PORTEC grade 3 subset and the Leuven, Zurich/Basel, and TCGA series, we applied Firth’s correction (24), owing to the absence of events in the *POLE-*mutant groups. For multivariable analysis of the additional series, we included disease stage as a covariable; although myometrial invasion, LVSI, and treatment were not included because of lack of data, we confirmed that omission of these variables from the PORTEC multivariable analyses did not alter estimates of RFS or CSS with *POLE* mutation (*P* = .93 and *P* = .87 respectively) (see Supplementary Methods, available online). Statistical analyses were performed using SPSS (SPSS Inc., Chicago, IL), Stata (StataCorp, College Station, TX) and R (http://www.r-project.org/). All *P* values were two-sided. A *P* value under .05 was considered statistically significant.

## Results

### Patient Characteristics and POLE Proofreading Mutations in PORTEC Studies

Demographic and clinicopathological characteristics of the PORTEC study participants in whom tumor *POLE* sequencing was successful are shown in Table 1. The majority (about 98%) of cancers in both studies were EECs. *POLE* exon 9 and 13 proofreading mutations were detected in 48 of 788 (6.1%) tumors from the combined PORTEC studies (Table 1), with similar distribution across study arms (11,12). With the exception of two tumors harboring a germline polymorphism of uncertain pathogenicity (rs150032060; c.1282G>A, p.Ala428Thr), detected in tumor-free myometrium in both cases and excluded from subsequent analyses, all mutations were recurrent substitutions at somatic mutational hotspot codons (Supplementary Table 7, available online) known to cause ultramutation (11,12). Apart from one neuroendocrine tumor, all *POLE* mutations occurred in endometrioid ECs. Analysis of 48 available preoperative curettings identified *POLE* mutations in all five cases in which they were detected in the subsequent hysterectomy, resulting in 100% concordance. Compared with *POLE* wild-type ECs, *POLE*-mutant tumors occurred in younger women (median age 63.5 vs 68.5 years, *P* < .001, *t* test), and were more commonly grade 3 (31.3% vs 12.7%, *P* < .001, χ^2^ test), though LVSI and deep (>50%) myometrial invasion were less frequent (0% vs 9.5%, *P* = .03, and 58.2% vs 71.9%, *P* = .045, respectively, χ^2^ test) (Table 1).

**Table 1.** Clinicopathological characteristics of patients in PORTEC studies according to *POLE* proofreading mutation

Demographic/ clinicopathological characteristic	PORTEC-1 (n = 412)		PORTEC-2 (n = 376)		Combined (n = 788)		*P* ^†^
	*POLE* wild-type (n = 386)	*POLE* mutant* (n = 26)	*POLE* wild-type (n = 354)	*POLE* mutant* (n = 22)	*POLE* wild-type (n = 740)	*POLE* mutant* (n = 48)	
	No. (%)	No. (%)	No. (%)	No. (%)	No. (%)	No. (%)	
Age, y							
Mean	67.0	59.8	70.1	67.9	68.5	63.5	<.001
Range	41–90	46–76	46–88	61–81	41–90	46–81	
<60	91 (23.6)	16 (61.5)	15 (4.2)	0 (0)	106 (14.3)	16 (33.3)	.002
60–70	152 (39.4)	4 (15.4)	173 (48.9)	12 (54.5)	325 (43.9)	16 (33.3)	
>70	143 (37.0)	6 (23.1)	166 (46.9)	10 (45.5)	309 (41.8)	16 (33.3)	
Tumor type							
EEC	380 (98.4)	26 (100)	343 (96.9)	21 (95.5)	723 (97.7)	47 (97.9)	.92
NEEC	6 (1.6)	0 (0)	11 (3.1)	1 (4.5)	17 (2.3)	1 (2.1)	
FIGO stage (1988)							
IB	150 (38.9)	13 (50)	27 (7.6)	4 (18.2)	177 (23.9)	17 (35.4)	.19
IC	236 (61.1)	13 (50)	283 (79.9)	16 (72.7)	519 (70.1)	29 (60.4)	
IIA	NA	NA	44 (12.4)	2 (9.1)	44 (5.9)	2 (4.2)	
Depth of invasion							
<50%	150 (38.9)	13 (50)	58 (16.4)	7 (31.8)	208 (28.1)	20 (41.7)	.045
>50%	236 (61.1)	13 (50)	296 (83.6)	15 (68.2)	532 (71.9)	28 (58.3)	
Grade							
1	264 (68.4)	16 (61.5)	279 (78.8)	12 (54.5)	543 (73.4)	28 (58.3)	.001
2	63 (16.3)	3 (11.5)	40 (11.3)	2 (9.1)	103 (13.9)	5 (10.4)	
3	59 (15.3)	7 (26.9)	35 (9.9)	8 (36.4)	94 (12.7)	15 (31.3)	
LVSI							
Absent	364 (94.3)	26 (100)	306 (86.4)	22 (100)	670 (90.5)	48 (100)	.03
Present	22 (5.7)	0 (0)	48 (13.6)	0 (0)	70 (9.5)	0 (0)	
Radiotherapy							
NAT	196 (50.8)	13 (50)	3 (0.8)	0 (0)	199 (26·9)	13 (27.1)	.98
EBRT	190 (49.2)	13 (50)	171 (48.3)	11 (50)	361 (48.8)	24 (50)	
VBT	NA	NA	180 (50.8)	11 (50)	180 (24.3)	11 (22.9)	
Chemotherapy							
No	386 (100.0)	26 (100.0)	354 (100.0)	22 (100.0)	740 (100.0)	48 (100.0)	1.0
Yes	0 (0.0)	0 (0.0)	0 (0.0)	0 (0.0)	0 (0.0)	0 (0.0)	

* *POLE* exon 9 or 13 proofreading exonuclease domain mutation (excludes two cases of confirmed germline variant rs150032060 [c.1282G>A, p.Ala428Thr], confirmed in tumor-free myometrium and previously shown not to associate with tumor ultramutation). EBRT = (pelvic) external beam radiotherapy; EEC = endometrioid endometrial cancer; LVSI = lymphovascular space invasion; NAT = no additional treatment; NEEC = nonendometrioid endometrial cancer; VBT = vault brachytherapy.

^†^ *P* values represent comparison of *POLE* wild-type and *POLE* proofreading mutant cohorts in the combined cohorts calculated by unpaired *t* test (age) or χ^2^ test (all other comparisons). All statistical tests were two-sided.

### Clinical Outcome by POLE Proofreading Mutation in PORTEC Studies

**Figure 1.**
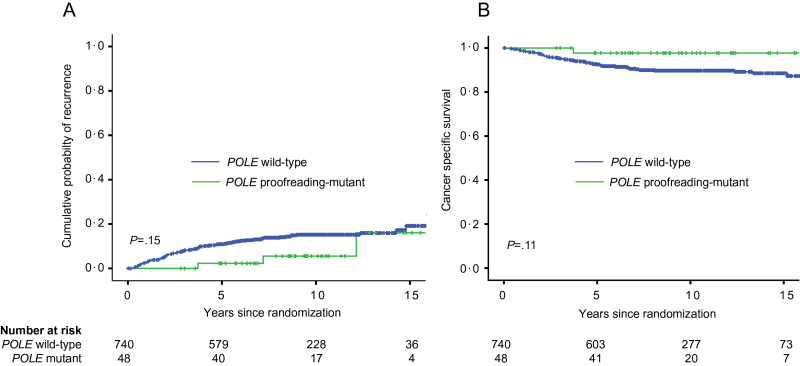
Cumulative probability of recurrence (**A**) and cancer-specific survival (**B**) according to *POLE* proofreading mutation in the combined PORTEC studies. *P* values are obtained by two-sided log-rank test.

We first examined the association of *POLE* proofreading mutation with EC recurrence. Three of 48 (6.2%) women with *POLE*-mutant tumors developed local or distant recurrence during study follow-up, compared with 104 of the other 740 (14.1%) patients (Figure 1A, Table 2). All three recurrences in the *POLE*-mutant cohort were distant metastases without locoregional relapse and occurred in women with grade 1 EECs and deep myometrial invasion, managed by observation, EBRT, and VBT, respectively. The univariable HR for recurrence-free survival (RFS) with tumor *POLE* proofreading mutation was 0.41 (95% CI = 0.13 to 1.28, *P* = .13), with little change following adjustment for known prognostic variables by Cox regression (HR = 0.43, 95% CI = 0.13 to 1.37, *P* = .15) (Figure 1A, Table 2; Supplementary Tables 3 and 5, available online). As many local EC recurrences are salvageable with therapy, we next examined whether cancer-specific survival (CSS) varied according to tumor *POLE* mutation. In women with *POLE*-mutant EC, there was one (2.3%) EC death during follow-up, compared with 72 (9.7%) in the rest of the study population. The unadjusted HR for CSS was 0.20 (95% CI = 0.03 to 1.46, *P* = .11), with minimal alteration following multivariable analysis (HR = 0.19, 95% CI = 0.03 to 1.44, *P* = .11) (Figure 1B, Table 2; Supplementary Tables 4 and 6, available online). Overall survival of women with *POLE*-mutant ECs was not statistically significantly greater than that of other patients by univariable or multivariable analysis (10-year OS = 76.2% vs 70.4%) (Table 2).

**Table 2.** Clinical outcome in PORTEC studies according to *POLE* proofreading mutation determined by univariable and multivariable analysis

Outcome	Events/total (%)	10-year, %	Univariable analysis		Multivariable analysis*	
			HR (95% CI)	*P* ^†^	HR (95% CI)	*P* ^†^
Recurrence						
*POLE* wild-type	104/740 (14.1)	15.2	0.41 (0.13 to 1.28)	.13	0.43 (0.13 to 1.37)	.15
*POLE* mutant^‡^	3/48 (6.2)	5.5				
Cancer-specific survival						
*POLE* wild-type	72/740 (9.7)	89.7	0.20 (0.03 to 1.46)	.11	0.19 (0.03 to 1.44)	.11
*POLE* mutant^‡^	1/48 (2.3)	97.7				
Overall survival						
*POLE* wild-type	248/740 (33.5)	70.4	0.69 (0.38 to 1.22)	.20	1.06 (0.59 to 1.92)	.85
*POLE* mutant^‡^	12/48 (25.0)	76.2				

* Multivariable Cox models include *POLE* mutation, age, nonendometrioid histology, tumor grade, and lymphovascular invasion. CI = confidence interval; HR = hazard ratio.

^†^ Calculated using Cox proportional hazards test. All statistical tests were two-sided.

^‡^ *POLE* exon 9 or 13 proofreading mutation.

**Figure 2.**
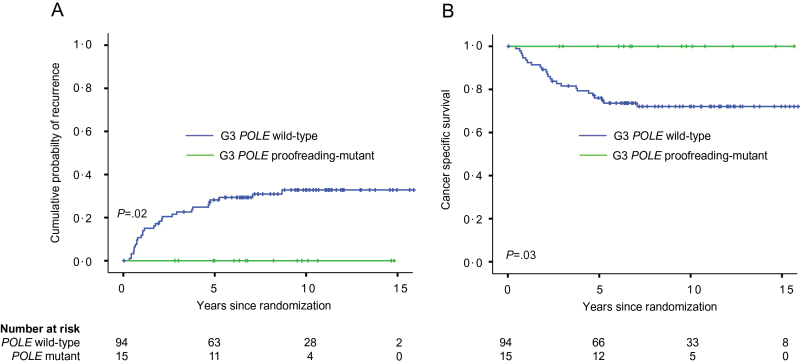
Cumulative probability of recurrence (**A**) and cancer-specific survival (**B**) according to *POLE* proofreading mutation in patients with grade 3 tumors in the combined PORTEC studies. *P* values are obtained by two-sided log-rank test.

The strong association of *POLE* mutations with high tumor grade (11) caused us to hypothesize that their apparent prognostic effect would be most evident in this group, who are commonly considered for treatment intensification. Of 109 patients with grade 3 tumors, there were no recurrences or cancer deaths in the 15 (13.7%) *POLE-*mutant case, compared with 29 (30.9%) relapses and 25 (26.6%) EC deaths in the remaining 94 women (Figure 2, A and B), reflected in statistically significantly improved RFS in univariable analysis (HR = 0.09, 95% CI = 0.001 to 0.66, *P* = .01) and, following adjustment for other prognostic variables in multivariable analysis (HR = 0.11, 95% CI = 0.001 to 0.84, *P* = .03) (Table 3; Supplementary Tables 8 and 9, available online). These results were essentially unchanged after limiting RFS analysis to the 97 grade 3 endometrioid ECs (univariable HR = 0.11, 95% CI = 0.001 to 0.78, *P* = .02; multivariable-adjusted HR = 0.12, 95% CI = 0.001 to 0.87, *P* = .03) (Supplementary Table 8, available online). Notably, *POLE* proofreading mutation was a stronger predictor of recurrence and EC death than all other prognostic variables examined in these analyses (Supplementary Tables 8 and 9, available online).

**Table 3.** Clinical outcome of patients with grade 3 tumors in PORTEC studies according to *POLE* proofreading mutation by univariable and multivariable analysis

Outcome	Events/total	10-year, %	Univariable analysis		Multivariable analysis*	
			HR (95% CI)	*P* ^†^	HR (95% CI)	*P* ^†^
Recurrence						
*POLE* wild-type	29/94	30.8	0.09 (0.001 to 0.66)	.01	0.11 (0.001 to 0.84)	.03
*POLE* mutant^‡^	0/15	0				
Cancer-specific survival						
*POLE* wild-type	25/94	73.4	0.11 (0.001 to 0.78)	.02	0.14 (0.001 to 1.01)	.05
*POLE* mutant^‡^	0/15	100				
Overall survival						
*POLE* wild-type	46/94	60	0.49 (0.18 to 1.36)	.17	0.78 (0.27 to 2.21)	.63
*POLE* mutant^‡^	4/15	73.3				

* Multivariable Cox models include *POLE* mutation, age, nonendometrioid histology, and lymphovascular invasion. CI = confidence interval; HR = hazard ratio.

^†^ Calculated using Cox proportional hazards test with Firth’s correction for analysis of recurrence and cancer-specific survival (because of absence of events in the *POLE*-mutant groups). All statistical tests were two-sided.

^‡^ *POLE* exon 9 or 13 proofreading mutation.

### Pooled Analysis With Additional EC Series

While the PORTEC analyses demonstrated a tendency for *POLE* proofreading-mutant ECs of all grades to improved outcome, the generally good prognosis of patients limited our ability to confirm this beyond the grade 3 subgroup. We therefore sought to support our results by analysis of two EC series from Leuven and Zurich/Basel, together with the published TCGA set, collectively comprising an additional 628 patients.

*POLE* proofreading mutations were detected in 33 (5.3%) case patients in the additional series (Supplementary Table 7, available online). As in the PORTEC studies, *POLE*-mutant tumors were more frequently high-grade (54.5% vs 32.4%, *P* = .013, χ^2^ test) and were generally of endometrioid histology, though three *POLE*-mutant mixed endometrioid/serous and one serous cancer were detected in the Leuven series (Supplementary Tables 10–12, available online).

**Figure 3.**
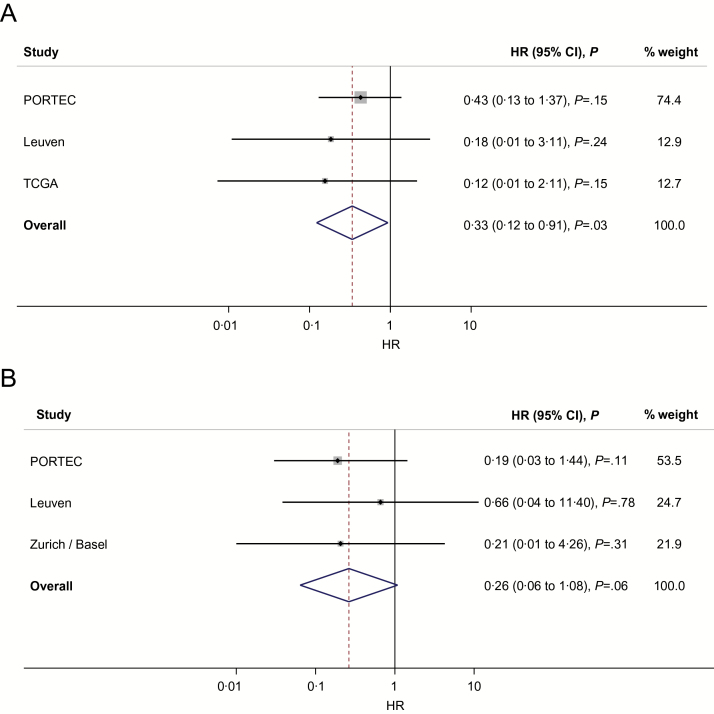
Pooled HRs for recurrence-free survival (**A**) and cancer-specific survival (**B**) of *POLE* proofreading-mutant endometrial cancer derived from the PORTEC studies and additional series. Results from multivariable analyses were combined by meta-analysis using Mantel-Haenszel weights to generate pooled, adjusted HRs and 95% confidence intervals. All statistical tests were two-sided. CI = confidence interval; HR = hazard ratio; TCGA = The Cancer Genome Atlas.

In the two series with RFS data—Leuven and TCGA—no *POLE*-mutant EC recurred with median follow-up of 28 months, compared with 31.6% and 19.0% of other tumors, respectively (Supplementary Figures 1A and 2, Supplementary Tables 13 and 14, available online). Similarly, for the two series in which CSS was documented—Leuven and Zurich/Basel (median follow-up 46 months)—there were no EC deaths in women with *POLE-*mutant tumors, compared with 18.6% and 10.8% of the remaining patients (Supplementary Figures 1B and 3, Supplementary Tables 13 and 15, available online). As anticipated given their sizes, no individual series demonstrated statistically significant differences in the RFS/CSS of *POLE*-mutant ECs by either univariable or multivariable analyses (Supplementary Tables 13–15, available online). We therefore combined multivariable-adjusted HRs from each series with those from the PORTEC studies by meta-analysis to generate pooled estimates for RFS and CSS according to *POLE* proofreading mutation (Figure 3, A and B). *POLE*-mutant ECs were associated with statistically significantly greater RFS compared with other tumors (HR = 0.33, 95% CI = 0.12 to 0.91, *P* = .03), with no evidence of heterogeneity among studies (*P* = .66), though the difference in CSS was not statistically significant using a two-tailed test (HR = 0.26, 95% CI = 0.06 to 1.08, *P* = .06). Interestingly, the only *POLE*-mutant tumors that recurred were early-stage, grade I ECs from the PORTEC cohorts (Supplementary Table 16, available online). To date, most *POLE* proofreading mutations have been detected in EECs (11,12,17), although *POLE*-mutant NEECs have been reported (11,25,26). Given the difficulty in histotyping high-grade ECs, we considered the possibility that the four *POLE*-mutant tumors reported as NEECs in the Leuven series may have been misclassified, thus biasing our results because of the poor prognosis of NEEC. We therefore confirmed that the estimate of RFS was essentially unchanged by repeating the meta-analysis after excluding *POLE* wild-type NEECs from all cohorts (HR = 0.34, 95% CI = 0.12 to 0.93, *P* = .04) (Supplementary Tables 5, 6, 13, and 14, available online).

## Discussion

By analyzing tumors from nearly 800 women in two large, randomly assigned controlled trials and pooling data with three independent series comprising over 600 additional patients, we have demonstrated that *POLE* proofreading-mutant ECs of all grades display excellent prognosis, independent of other known prognostic factors. *POLE*-mutant tumors have a risk of recurrence approximately one third that of other ECs, and a relative risk of cancer death that appears even lower, though the latter finding was not statistically significant in our analysis. This is despite a strong association of *POLE* proofreading mutations with high tumor grade—a characteristic that predicts a high risk of metastases in early EC (27). Indeed, in the PORTEC studies, no *POLE*-mutant grade 3 EC recurred, compared with 30.9% of grade 3 tumors in the rest of this subgroup, consistent with a recent report, which combined high-grade ECs from the TCGA series with an additional, small retrospective series (17). The role of adjuvant chemotherapy in patients with high-risk grade 3 tumors is currently under investigation, and as systemic therapy was not used in PORTEC-1 or -2 these data suggest that patients with *POLE* proofreading-mutant EC may be unlikely to benefit from such treatment. Similarly, the absence of locoregional recurrence of *POLE*-mutant ECs was observed across all arms of the PORTEC studies, including the NAT arm of PORTEC-1. While further studies are required before firm conclusions can be drawn on the implications of *POLE* proofreading mutations for postoperative treatment in EC, our results suggest that minimization of adjuvant therapy for *POLE*-mutant ECs localized to the uterus may be worthy of investigation. They also suggest *POLE* proofreading mutation should be considered for inclusion in cancer gene panels used in EC, as it may improve prognostication, particularly for grade 3 tumors.

While highlighting the strengths of using high-quality clinical trial sample banks for biomarker research (18), our study has limitations. The challenge of confirming even a strong effect of a marker with modest (<10%) frequency in a population with relatively favorable prognosis meant that we used additional EC series to confirm the improved RFS of *POLE*-mutant ECs. While acknowledging the limitations of sets containing a mixture of histological subtypes and stages, with limited follow-up and lacking comprehensive treatment data (18) the similar results from each following multivariable analysis suggests that our results are unlikely to be because of chance or confounding by inclusion of NEECs in our analyses. However, as highlighted above, it will be important to confirm the favorable prognosis of *POLE*-mutant ECs in further independent series, and particularly in tumors of advanced stages. It should also be noted that as a single biomarker survey of *POLE* hotspot exons in ECs of predominantly endometrioid histology, we are presently unable to determine the effect of the about 10% of pathogenic variants outside exons 9 and 13, (11,12) or whether the effect of *POLE* mutation varies according to tumor molecular subtypes. Both questions are likely to be addressed by future studies.

*POLE* proofreading-mutant cancers are a molecularly distinct group of tumors with a striking mutation burden and distinctive mutation signature (11–13). Whether these characteristics contribute to their favorable prognosis awaits confirmation. Study of mutator polymerases in yeast has confirmed the existence of a mutational threshold, which, if exceeded, results in decreased viability because of lethal mutations in essential genes prior to cell division (28). It will be of interest to determine whether the dramatic increase in mutation rate in *S. cerevisiae* caused by the corresponding substitution to human *POLE* p.Pro286Arg (29) approaches this error threshold. Similarly, analysis of the burden of deleterious mutations accumulated in *POLE* proofreading-mutant cancers may provide insights into their behavior (30).

Over the last two years, we and others have shown that somatic mutations in the proofreading domain of *POLE* occur in several human tumors (10–12,20,31). We now demonstrate that, despite a strong association with high grade, *POLE* proofreading-mutant ECs have a favorable prognosis. While the frequency of *POLE* mutation in EC is modest, it is worth noting that it is broadly similar to that of many novel molecular aberrations recently discovered by TCGA and other sequencing efforts. As most common cancer variants are currently not actionable, similar analyses of these modest-frequency (5% to 10%) molecular subgroups are likely to be essential if we are to realize the ambition of personalized cancer medicine during the next decade.

## Funding

This work was supported by the Dutch Cancer Society Grant (UL2012-5719), Cancer Research UK (C6199/A10417), the European Union Seventh Framework Programme (FP7/207- 2013) grant 258236 collaborative project SYSCOL, the Oxford National Institute for Health Research (NIHR) Comprehensive Biomedical Research Centre, and core funding to the Wellcome Trust Centre for Human Genetics from the Wellcome Trust (090532/Z/09/Z). DNC is funded by a Health Foundation/Academy of Medical Sciences Clinician Scientist fellowship and has previously received salary support from an NIHR Academic Clinical Lecturer award and funding from the Oxford Cancer Research Centre. PJW is funded by a grant of the Baugarten Foundation, Zurich, Switzerland. FA is funded by FWO-Flanders.

## Supplementary Material

Supplementary Data

## Notes

The funders had no role in the design, analysis, interpretation, or manuscript preparation for this biomarker study.

The authors would like to thank the many individuals who participated in the PORTEC studies and the numerous institutions and their staff who have supported recruitment. We also thank Michelle Osse, Enno Dreef, Martina Storz, Susanne Dettwiler, Annette Bohnert, Chrissie Fieger, Peggy Tzscheetzsch, and Sonja Brun-Schmid for excellent technical assistance, the Tissue Biobank of the University Hospital Zurich for providing samples and Holger Moch and Rosmarie Caduff for constructing the tissue microarrays for the Zurich/ Basel series, and the patients who contributed samples. We thank the research staff of the Vesalius Research Center, FWO-Flanders where Frédéric Amant is a senior scientist, and the patients who provided samples for the Leuven series. We are also grateful to Victoria Strauss (Centre for Statistics in Medicine, Oxford) for statistical advice. The results published here include data generated by The Cancer Genome Atlas project funded by the National Cancer Institute and the National Institutes of Health (cancergenome.nih.gov); we would like to thank the many patients and researchers who contributed to this study.
