# Supplementary material for: Prognostic Significance of POLE Proofreading Mutations in Endometrial Cancer
Source: J Natl Cancer Inst. 2014 Dec 12;107(1):dju402. doi: 10.1093/jnci/dju402 (PMC4301706; doi:10.1093/jnci/dju402)
Supplement: Supplementary Data [file supp_107_1_dju402__index.html]

Prognostic Significance of POLE Proofreading Mutations in Endometrial Cancer — Supplementary Data 

# Prognostic Significance of *POLE* Proofreading Mutations in Endometrial Cancer

## Supplementary Data

Data files

**Files in this Data Supplement:**

- Supplementary Data - Supplementary Data
