# Supplementary material for: Prognostic Significance of POLE Proofreading Mutations in Endometrial Cancer
Source: J Natl Cancer Inst. 2014 Dec 12;107(1):dju402. doi: 10.1093/jnci/dju402 (PMC4301706; doi:10.1093/jnci/dju402)
Supplement: Supplementary Data [file supp_dju402_14_0249R1_Tomlinson_supp_mat_102914.docx]

**Prognostic significance of *POLE* proofreading mutations in endometrial cancer.**

David N Church, Ellen Stelloo, Remi A Nout, Nadejda Valtcheva, Jeroen Depreeuw, Natalja ter Haar, Aurelia Noske, Frederic Amant, Ian PM Tomlinson, Peter J Wild, Diether Lambrechts, Ina M Jürgenliemk-Schulz, Jan. J Jobsen, Vincent THBM Smit, Carien L Creutzberg, Tjalling Bosse

**Supplementary Methods**

**PORTEC studies.** Full details of the PORTEC-1 and PORTEC-2 clinical trials are provided in the original publications (1,2). Inclusion criteria for PORTEC-1 were histologically proven endometrial adenocarcinoma (including adenocarcinoma with squamous features, adenosquamous carcinoma, papillary serous carcinoma, and clear-cell carcinoma), of intermediate risk, defined as postoperative FIGO stage I, grade 1 with deep (>50%) myometrial invasion, grade 2 with any depth of invasion, or grade 3 with superficial (<50%) invasion. 662/714 (92.7%) of tumors were endometrioid endometrial cancers (EECs). Tumor subtype and grade were confirmed by central pathology review in 567 (79.4%) cases.

Eligibility criteria for PORTEC-2 were endometrial adenocarcinoma of high-intermediate risk, defined as: (i) age greater than 60 years and stage IC grade 1 or 2 disease, or stage IB grade 3 disease; or (ii) patients of any age with stage IIA disease (apart from grade 3 with greater than 50% myometrial invasion). Patients whose cancers had serous or clear cell histology were excluded. Central pathology review was performed in 86% cases, following which 12 tumors were reclassified as of non-endometrioid histology.

**Leuven series.** Details of the Leuven prospective series have been previously reported (3). Pathological analysis and grading were performed by a single, experienced gynaecological pathologist according to the WHO classification of Tumors of Female Reproductive Organs. Clinical information was collected by trained researchers. Patients were managed by TAH-BSO, with pelvic lymphadenectomy in cases of grade 1-2 tumors with diameter > 2 cm or 50% myometrial invasion, or grade 3 EECs and NEECs. Patients were followed up every 3 months for the first 2 years, every 6 months from 2-5 years and yearly afterwards. Follow-up data including in details of RFS, CSS and OS were extracted from electronic patient files.

**Zurich/Basel series.** Details of the Basel/Zurich series have been previously reported (4-6). Tumor blocks were collected retrospectively from patients treated in academic centres and outpatient clinics. Staging was according to the AJCC TNM system (7^th^ edition), and classified by two independent histopathologists with the aid of IHC. Patients with localized disease were treated by TAH-BSO (with or without pelvic and para-aortic lymphadenectomy). Adjuvant intra-vaginal radiation therapy was given postoperatively in cases of myometrial invasion or grade 3 tumor. Follow-up information was obtained from the participating centres and from the Cancer Registries of Basel and Zurich.

**TCGA series.** Details of the TCGA EC series have been previously reported (7). Patients were recruited from participating centres, and tumors staged according to the AJCC system. Whole exome sequencing was performed on 248 cases. Treatment and follow up were at the discretion of the treating clinician; of the overall population, 19% received adjuvant RT, 10% adjuvant chemotherapy, and 14% adjuvant chemoradiotherapy; in 51% of cases, the postoperative treatment delivered was not known.

**DNA extraction.** In the PORTEC studies, DNA was extracted from 0.6mm tissue punches taken from areas containing ≥70% tumor in FFPE blocks, with exception of 14 cases with tumor size of <0.6mm, or insufficient fraction of tumor cells, where DNA was extracted following manual microdissection by standard methods. Similar methodology was used to extract tumor DNA in the Zurich/Basel series, while in Leuven series, DNA was extracted from fresh-frozen tissue using DNeasy Blood & Tissue Kit (Qiagen), following confirmation of adequate tumor cellularity using H&E slides on FFPE material.

**PCR and sequencing reactions.** PCR primers for sequencing of *POLE* exons 9 and 13 are shown in Table S1. PCR was performed using 10ng of template DNA with Qiagen multiplex PCR kit with Q solution in a 15μL reaction volume, with primers at 0.4μM final concentration and proportions of other reagents according to the manufacturer’s recommendations. Reaction conditions were as follows; 95°C denaturation for 15 minutes; 94°C melt for 45 seconds, 55°C annealing for 90 seconds and 72°C extension for 45 seconds repeated for 38 cycles; and final 72°C extension for 10 minutes. PCR products were confirmed by gel electrophoresis and cleaned up with Exosap-IT (Affymetrix) as per manufacturer’s instructions. BDT (Life technologies) sequencing was performed according to manufacturer’s recommendations using the forward primer for sequencing of *POLE* exon 9 and reverse primer for *POLE* exon 13.

PORTEC. PCR screening of *POLE* exons 9 and 13 was successful in 412/434 (94.9%) and 376/398 (95.4%) cases respectively from the PORTEC-1 and -2 studies. There was no significant difference between the biomarker study and overall populations in the proportion of cases with grade 3 tumors (13.7% vs. 13.3%, *P*=0.84) or disease stage (*P*=0.07).

Leuven. Of 187 fresh frozen samples analysed in the Leuven set, PCR screening was successful in 183 (97.9%) cases. Of these, 13 (7.1%) were excluded due to non-endometrial histology (n=7, 3.8%), missing data (n=2, 1.1%), and insufficient follow up (n=4, 2.2%). There was no significant difference between the biomarker and overall populations in the proportion of cases with grade 3 tumors (54.3% vs. 55.2%, *P*=0.92) or stage III/IV disease (30.4% vs. 31.2%, *P*=0.91).

TCGA. Data were downloaded from cBioportal (<http://www.cbioportal.org/public-portal/>). Of 248 cases with whole-exome sequence data, 19 were excluded due to absence of outcome data (n=17, 6.9%) or lack of tumor grading (n=2, 0.8%). There was no significant difference between the analysed and overall populations in the proportion of cases with grade 3 tumors (39.1% vs. 37.1%, *P*=0.71) or stage III/IV disease (23.0% vs. 21.0%, *P*=0.66).

Zurich/Basel. Of 267 cases from the Zurich/Basel set in whom outcome data and tumor DNA were available, PCR screening was successful in 260 cases (97.4%). 31 (11.9%) cases were excluded from multivariable analysis due to absence of data on tumor grade (n=30, 11.5%) or grade and stage (n=1, 0.4%). There was no significant difference between the biomarker and overall populations in the proportion of cases with grade 3 tumors (18.0% vs. 14.4%, *P*=0.24) or stage III/IV disease (18.6% vs. 18.5%, *P*=1.0).

**Statistical Analyses**

All informative subjects were used for statistical analyses, and subjects in whom data were absent were excluded. Baseline clinicopathological and molecular variables were compared using Fisher’s exact or Chi-square test for categorical variables, and Student’s t-test for continuous variables. Analyses performed in this biomarker study are listed in Table S2 in accordance with published guidelines. All statistical tests were two-sided.

For analysis of clinical outcome, we used multivariable Cox proportional hazards models to minimize confounding from prognostic factor imbalance between *POLE* proofreading-mutant and wild-type groups in all datasets. For the PORTEC studies, 784 patients had complete data and were informative for Cox regression analysis. In accordance with published guidelines (8,9), for the PORTEC analyses, we first examined the effect of addition of *POLE* proofreading mutation to a Cox model containing all standard prognostic factors for which data were available (Table S3, S4). For the final Cox models reported, variables associated with recurrence at a significance level of *P*<0.1 by univariable analysis (age, non-endometrioid histology, grade and lymphovascular invasion) were included in the initial model. With the exception of non-endometrioid histology, all four variables retained independent significance at *P*<0.1 following the addition of *POLE* EDM status to the model and were used for the final analyses (Tables S5, S6). For the grade 3 subgroup in PORTEC, and the non-PORTEC series, we used the Firth correction (10,11) owing to the absence of events in the *POLE* proofreading-mutant cohorts. Although we were unable to include myometrial invasion, LVSI and treatment in multivariable analyses of the additional series, we confirmed that their omission from the ‘standardised’ PORTEC Cox models containing all prognostic variables (Tables S3,S4) did not significantly alter the estimates of the effect of *POLE* mutation on either RFS (HR=0.39, 95% CI 0.12-1.23 vs. HR=0.46, 95% CI 0.14-1.47, *P*=0.88) or CSS (HR=0.18, 95% CI 0.03-1.29 vs. HR=0.20, 95% CI 0.03-1.46, *P=*0.93) (12). For these analyses, confidence intervals and *P* values were calculated by the Wald method using the Coxphf function in R (http://www.r-project.org/). Multivariable-adjusted hazard ratios were pooled by meta-analysis using the metan command in Stata.

**References**

1. Creutzberg CL, van Putten WL, Koper PC, Lybeert ML, Jobsen JJ, Wárlám-Rodenhuis CC, et al. Surgery and postoperative radiotherapy versus surgery alone for patients with stage-1 endometrial carcinoma: multicentre randomised trial. PORTEC Study Group. *Lancet*. 2000 22;355(9213):1404-11.

2. Nout RA, Smit VT, Putter H, Jurgenliemk-Schulz IM, Jobsen JJ, Lutgens LC, et al. Vaginal brachytherapy versus pelvic external beam radiotherapy for patients with endometrial cancer of high-intermediate risk (PORTEC-2): an open-label, non-inferiority, randomised trial. *Lancet Oncology*. 2010;375(9717):816-23.

3. Garcia-Dios DA, Lambrechts D, Coenegrachts L, Vandenput I, Capoen A, Webb PM, et al. High-throughput interrogation of PIK3CA, PTEN, KRAS, FBXW7 and TP53 mutations in primary endometrial carcinoma. *Gynecol Oncol*. 2012:1-8.

4. Dellas A, Jundt G, Sartorius G, Schneider M, Moch H. Combined PTEN and p27kip1 protein expression patterns are associated with obesity and prognosis in endometrial carcinomas. *Clin Cancer Res*. 2009 1;15(7):2456-62

5. Lebeau A, Grob T, Holst F, Seyedi-Fazlollahi N, Moch H, Terracciano L, et al. Oestrogen receptor gene (ESR1) amplification is frequent in endometrial carcinoma and its precursor lesions. *J Pathol*. 2008;216(2):151-7

6. Wild PJ, Ikenberg K, Fuchs TJ, Rechsteiner M, Georgiev S, Fankhauser N, et al. p53 suppresses type II endometrial carcinomas in mice and governs endometrial tumor aggressiveness in humans. *EMBO Mol Med.* 2012;4(8):808-24.

7. Kandoth C, Schultz N, Cherniack AD, Akbani R, Liu Y, Shen H, et al. Integrated genomic characterization of endometrial carcinoma. *Nature.* 2013;497(7447):67-73.

8. McShane LM, Altman DG, Sauerbrei W, Taube SE, Gion M, Clark GM, et al. Reporting Recommendations for Tumor Marker Prognostic Studies (REMARK). *Journal of the National Cancer Institute*. 2005;97(16):1180-4.

9. Altman DG., McShane, L. M., Sauerbrei, W. and Taube, SE. Reporting Recommendations for Tumor Marker Prognostic Studies (REMARK): explanation and elaboration. *PLoS medicine* 9, e1001216: (2012).

10. Heinze G, Schemper M. A solution to the problem of monotone likelihood in Cox regression. *Biometrics*. 2001;57(1):114-9.

11. Heinze G,. & Dunkler D. Avoiding infinite estimates of time-dependent effects in small-sample survival studies. *Stat Med, 2008;* 27, 6455-6469

12. Clogg C, Petkova E, Haritou A. Statistical Methods for Comparing Regression Coefficients Between Models. *The American Journal of Sociology*, 1995; 100(5): 1261-1293

**Supplementary Table 1. *POLE* exon 9 and 13 sequencing PCR primer details**

| **Target (residues)** | **Primer name** | **Primer sequence** | **Primer used for sequencing** |
| --- | --- | --- | --- |
| Exon 9  (268-303) | *POLE*-Ex9Fw | 5′- tgcttattttgtccccacag-3′ | Forward |
|  | *POLE*-Ex9Rv | 5′- tacttcccagaagccacctg-3′ |  |
| Exon 13  (410-445) | *POLE-*Ex13Fw | 5′- tctgttctcattctccttccag-3′ | Reverse |
|  | *POLE*-Ex13Rv | 5′- cgggatgtggcttacgtg-3′ |  |

**Supplementary Table 2. Analyses performed and reported in this study**

| **Comparison** | **Population** | **Primary endpoint** | **Secondary endpoints** | **Analysis** | **Reported** |
| --- | --- | --- | --- | --- | --- |
| *POLE* proofreading-mutant vs. *POLE* wild-type | Combined PORTEC 1/2 studies, all patients* | RFS | CSS, OS | Log rank *P,* univariable and multivariable adjusted HR | Main text, Tables 2, S3-S6, Figure 1, |
| *POLE* proofreading-mutant vs. *POLE* wild-type | Combined PORTEC 1/2 studies, grade 3 subgroup* | RFS | CSS | Log rank *P,* univariable and multivariable adjusted HR | Main text, Figure 2, Tables 3,S8,S9 |
| *POLE* proofreading-mutant vs. *POLE* wild-type | Leuven series, all patients* | RFS | CSS | Log rank *P*, Univariable and multivariable adjusted HR | Table S13, Figure S1 |
| *POLE* proofreading-mutant vs. *POLE* wild-type | TCGA series, all patients* | RFS | CSS | Log rank *P* , Univariable and multivariable adjusted HR | Table S14, Figure S2 |
| *POLE* proofreading-mutant vs. *POLE* wild-type | Zurich/Basel series, all patients* | CSS | NA | Log rank *P*, Univariable and multivariable adjusted HR | Table S15, Figure S3 |
| *POLE* proofreading-mutant vs. *POLE* wild-type | Pooled PORTEC, Leuven & TCGA series | RFS | NA | Weighted HR from pooled multivariable analyses | Main text, Figure 3A |
| *POLE* proofreading-mutant vs. *POLE* wild-type | Pooled PORTEC, Leuven & Zurich/Basel series | CSS | NA | Weighted HR from pooled multivariable analyses | Main text, Figure 3B |

*Analyses include both tumors of all histologies and endometrioid endometrial cancers (EECs) only

**Supplementary Table 3. Cox regression model for recurrence-free survival in PORTEC-1 and -2 demonstrating effect of addition of *POLE* proofreading mutation to model of standard prognostic variables**

| **Variable** | **HR (95% CI)** | ***P**** |
| --- | --- | --- |
| *Base model* |  |  |
| NEEC vs. EEC | 1.82 (0.76 – 4.33) | 0.18 |
| Tumor grade |  |  |
| Grade 2 vs. grade 1 | 2.30 (1.42 – 3.71) | 0.001 |
| Grade 3 vs. grade 1 | 3.41 (2.11 – 5.52) | <0.0001 |
| Myometrial invasion (>50% vs. <50%) | 1.48 (0.92 – 2.37) | 0.11 |
| LVSI (present vs. absent) | 2.28 (1.40 – 3.69) | 0.001 |
| Age (continuous) | 1.04 (1.01 – 1.06) | 0.004 |
| Treatment |  |  |
| EBRT vs. NAT | 0.61 (0.39 – 0.95) | 0.03 |
| VBT vs. NAT | 0.84 (0.50 – 1.40) | 0.50 |
| *Base model with addition of POLE mutation* |  |  |
| *POLE* mutation^†^ | 0.46 (0.14 – 1.47) | 0.19 |

* *P* value calculated by two-sided Cox proportional hazards test.

^†^ *POLE* proofreading exonuclease domain mutations detected from sequencing exons 9 and 13 only (contain ~80% pathogenic variants)

EEC – endometrioid endometrial cancer, NEEC – non-endometrioid endometrial cancer. LVSI – lymphovascular space invasion, EBRT – external beam radiotherapy, VBT – vault brachytherapy, NAT – no additional treatment

**Supplementary Table 4. Cox regression model for cancer-specific survival in PORTEC-1 and -2 demonstrating effect of addition of *POLE* proofreading mutation to model of standard prognostic variables**

| **Variable** | **HR (95% CI)** | ***P**** |
| --- | --- | --- |
| *Base model* |  |  |
| NEEC vs. EEC | 1.70 (0.65 – 4.44) | 0.28 |
| Tumor grade |  |  |
| Grade 2 vs. grade 1 | 2.43 (1.34 – 4.40) | 0.003 |
| Grade 3 vs. grade 1 | 5.43 (3.11 – 9.49) | <0.001 |
| Myometrial invasion (>50% vs. <50%) | 1.80 (1.01 – 3.19) | 0.045 |
| LVSI (present vs. absent) | 1.93 (1.08 – 3.45) | 0.03 |
| Age (continuous) | 1.04 (1.01 – 1.07) | 0.02 |
| Treatment |  | 0.90 |
| EBRT vs. NAT | 1.14 (0.65 – 1.20) | 0.65 |
| VBT vs. NAT | 1.14 (0.59 – 2.28) | 0.70 |
| *Base model with addition of POLE mutation* |  |  |
| *POLE* mutation^†^ | 0.2 (0.03 – 1.46) | 0.11 |

* *P* value calculated by two-sided Cox proportional hazards test.

^†^ *POLE* proofreading exonuclease domain mutations detected from sequencing exons 9 and 13 only (contain ~80% pathogenic variants)

EEC – endometrioid endometrial cancer, NEEC – non-endometrioid endometrial cancer. LVSI – lymphovascular space invasion, EBRT – external beam radiotherapy, VBT – vault brachytherapy, NAT – no additional treatment

| **Variable** | **HR (95% CI)** | ***P**** |
| --- | --- | --- |
| *All tumors (n=788)* |  |  |
| Tumor grade |  |  |
| Grade 2 vs. grade 1 | 2.52 (1.55–4.08) | <0.001 |
| Grade 3 vs. grade 1 | 3.47 (2.20 – 5.47) | <0.001 |
| LVSI (present vs. absent) | 2.16 (1.32 – 3.54) | 0.002 |
| Age (continuous) | 1.04 (1.01–1.06) | 0.003 |
| *POLE* mutation | 0.43 (0.13 – 1.37) | 0.15 |
| *EECs only (n=770)* |  |  |
| *POLE* mutation^†^ | 0.44 (0.14 – 1.42) | 0.17 |

**Supplementary Table 5. Final multivariable Cox models for recurrence-free survival including *POLE* proofreading mutation using all PORTEC cases and limited to endometrioid ECs only**

* *P* value calculated by two-sided Cox proportional hazards test.

^†^ *POLE* proofreading exonuclease domain mutations detected from sequencing exons 9 and 13 only (contain ~80% pathogenic variants)

EEC – endometrioid endometrial cancer, LVSI – lymphovascular space invasion.

**Supplementary Table 6. Final multivariable Cox models for cancer-specific survival including *POLE* proofreading mutation using all PORTEC cases and limited to endometrioid ECs only**

| **Variable** | **HR (95% CI)** | ***P**** |
| --- | --- | --- |
| *All tumors (n=788)* |  |  |
| Tumor grade |  |  |
| Grade 2 vs. grade 1 | 2.69 (1.47–4.91) | <0.001 |
| Grade 3 vs. grade 1 | 5.03 (2.97–8.51) | <0.001 |
| LVSI (present vs. absent) | 2.10 (1.16–3.78) | 0.01 |
| Age (continuous) | 1.04 (1.01–1.08) | 0.006 |
| *POLE* mutation | 0.19 (0.03–1.44) | 0.11 |
| *EECs only (n=770)* |  |  |
| *POLE* mutation^†^ | 0.21 (0.03 – 1.50) | 0.12 |

* *P* value calculated by two-sided Cox proportional hazards test.

^†^ *POLE* proofreading exonuclease domain mutations detected from sequencing exons 9 and 13 only (contain ~80% pathogenic variants)

EEC – endometrioid endometrial cancer, LVSI – lymphovascular space invasion.

**Supplementary Table 7. *POLE* proofreading mutations detected in PORTEC-1/2 and additional series**

|  |  | **PORTEC 1 & 2**  **(n=788)** | | **Leuven**  **(n=170)** | | **Zurich/Basel**  **(n=229)** | | **TCGA**  **(n=229)** | |
| --- | --- | --- | --- | --- | --- | --- | --- | --- | --- |
| Nucleotide change | Amino acid change | No. | % | No. | % | No. | % | No. | % |
| c.857C>G | p.Pro286Arg | 31 | 3.9 | 5 | 2.9 | 5 | 2.2 | 8 | 3.5 |
| c.890C>T | p.Ser297Phe | 2 | 0.3 | 0 | 0 | 0 | 0 | 1 | 0.4 |
| c.890C>A | p.Ser297Tyr | 1 | 0.1 |  |  |  |  |  |  |
| c.895A>G* | p.Met299Va* | 0 | 0 | 1 | 0.6 | 0 | 0.0 | 0 | 0 |
| c.1231G>C  / c.1231G>T | p.Val411Leu | 14 | 1.8 | 3 | 1.8 | 1 | 0.4 | 5 | 2.2 |
| c.1270C>A | p.Leu424Ile | 0 | 0 | 0 | 0 | 0 | 0 | 1 | 0.4 |
| c.1270C>G | p.Leu424Val | 0 | 0 | 0 | 0 | 0 | 0 | 1 | 0.4 |
| c.1331T>A | p.Met444Lys | 0 | 0 | 0 | 0 | 0 | 0 | 1 | 0.4 |
| Other (non-exon 9/13) | | NA | NA | NA | | NA | | 1 | 0.4 |
| Total | | 48 | 6.1 | 9 | 5.3 | 6 | 2.6 | 18 | 7.9 |

*Variant not previously reported affecting residue absolutely conserved in Pol ε orthologues close to hotspot codon 297 and predicted to be deleterious by mutation assessor (score 2.35) and SIFT (score 0.00). “Other” indicates non-exon 9/13 variants detected by whole exome sequencing and associated with ultramutation in TCGA analysis.

**Supplementary Table 8. Multivariable Cox model for recurrence-free survival of PORTEC grade 3 tumor subgroup including *POLE* proofreading mutation and standard prognostic variables**

| **Variable** | **HR (95% CI)** | ***P**** |
| --- | --- | --- |
| *All grade 3 tumors (n=109)* |  |  |
| NEEC vs. EEC | 1.96 (0.68–4.81) | 0.20 |
| LVSI (present vs. absent) | 1.13 (0.25–2.87) | 0.82 |
| Myometrial invasion (>50% vs. <50%) | 1.86 (0.89–3.95) | 0.10 |
| Age (continuous) | 1.01 (0.96–1.05) | 0.83 |
| *POLE* mutation | 0.11 (0.001–0.84) | 0.03 |
| *Grade 3 EECs only (n=97)* |  |  |
| *POLE* mutation^†^ | 0.12 (0.001–0.87) | 0.03 |

* *P* value calculated by two-sided Cox proportional hazards test with Firth correction

^†^ *POLE* proofreading exonuclease domain mutations detected from sequencing exons 9 and 13 only (contain ~80% pathogenic variants)

NEEC – non-endometrioid endometrial cancer, EEC – endometrioid endometrial cancer, LVSI – lymphovascular space invasion.

**Supplementary Table 9. Multivariable Cox model for cancer-specific survival of PORTEC grade 3 tumor subgroup including *POLE* proofreading mutation and standard prognostic variables**

| **Variable** | **HR (95% CI)** | ***P**** |
| --- | --- | --- |
| *All grade 3 tumors (n=109)* |  |  |
| NEEC vs. EEC | 1.84 (0.56–4.86) | 0.28 |
| LVSI (present vs. absent) | 1.01 (0.27–2.82) | 0.98 |
| Myometrial invasion (>50% vs. <50%) | 1.65 (0.75–3.66) | 0.21 |
| Age (continuous) | 1.02 (0.97–1.08) | 0.42 |
| *POLE* mutation | 0.14 (0.001–1.01) | 0.05 |
| *Grade 3 EECs only (n=97)* |  |  |
| *POLE* mutation^†^ | 0.15 (0.001–1.10) | 0.07 |

* *P* value calculated by two-sided Cox proportional hazards test with Firth correction

^†^ *POLE* proofreading exonuclease domain mutations detected from sequencing exons 9 and 13 only (contain ~80% pathogenic variants)

NEEC – non-endometrioid endometrial cancer, EEC – endometrioid endometrial cancer, LVSI – lymphovascular space invasion.

**Supplementary Table 10. Demographic and clinicopathological characteristics of Leuven series according to *POLE* proofreading mutation**

|  | **Leuven**  (N=170) | | | |  |
| --- | --- | --- | --- | --- | --- |
| **Demographic / clinicopathological characteristic** | ***POLE* wild-type**  N=161 (94.7%) | | ***POLE* mutant***  N=9 (5.3%) | |  |
|  | N | % | N | % | *P^†^* |
| **Age, years** |  |  |  |  |  |
| Median (range) | 70 (36 – 93) | | 57 (45 – 81) | | 0.06 |
| **Tumor type** |  |  |  |  |  |
| EEC | 103 | 64.0% | 5 | 55.6% | 0.75 |
| NEEC^§^ | 58 | 36.0% | 4 | 44.4% |  |
| **FIGO stage** |  |  |  |  |  |
| I | 92 | 57.1% | 6 | 66.7% | 0.50 |
| II | 18 | 11.2% | 1 | 11.1% |  |
| III | 30 | 18.6% | 0 | 0.0% |  |
| IV | 21 | 13.0% | 2 | 22.2% |  |
| **Grade** |  |  |  |  |  |
| 1 | 41 | 25.5% | 2 | 22.2% | 0.73 |
| 2 | 32 | 19.9% | 1 | 11.1% |  |
| 3 | 88 | 54.7% | 6 | 66.7% |  |

* *POLE* proofreading exonuclease domain mutations detected from sequencing exons 9 and 13 only (contain ~90% pathogenic variants).

*^†^ P* values represent comparison of *POLE* wild-type and *POLE* proofreading mutant groups calculated by unpaired t-test (age), Fisher’s exact test (tumor type), or χ^2^ test (other). All statistical tests were two-sided.

EEC – endometrioid endometrial cancer, NEEC – non-endometrioid endometrial cancer.

**Supplementary Table 11. TCGA.** **Demographic and clinicopathological characteristics of TCGA series according to *POLE* proofreading mutation**

|  | **TCGA**  (N=229) | | | |  |
| --- | --- | --- | --- | --- | --- |
| **Demographic / clinicopathological characteristic** | ***POLE* wild-type**  N=211 (92.1%) | | ***POLE* mutant***  N=18 (7.9%) | |  |
|  | N | % | N | % | *P^†^* |
| **Age, years** |  |  |  |  |  |
| Median (range) | 63 (34-90) | | 57 (33-87) | | 0.06 |
| **Tumor type** |  |  |  |  |  |
| EEC | 170 | 80.6% | 18 | 100.0% | 0.049 |
| NEEC | 41 | 19.4% | 0 | 0.0% |  |
| **FIGO stage** |  |  |  |  |  |
| I | 156 | 73.9% | 13 | 72.2% | 0.74 |
| II | 11 | 5.2% | 1 | 5.6% |  |
| III | 34 | 16.1% | 4 | 22.2% |  |
| IV | 10 | 4.7% | 0 | 0.0% |  |
| **Grade** |  |  |  |  |  |
| 1 | 65 | 30.8% | 6 | 33.3% | 0.61 |
| 2 | 69 | 32.4% | 4 | 22.2% |  |
| 3 | 77 | 36.5% | 8 | 44.4% |  |

**^*^** *POLE* proofreading exonuclease domain mutations detected from whole exome sequencing.

*^†^ P* values represent comparison of *POLE* wild-type and *POLE* proofreading mutant groups calculated by unpaired t-test (age), Fisher’s exact test (tumor type), or χ^2^ test (other). All statistical tests were two-sided.

EEC – endometrioid endometrial cancer, NEEC – non-endometrioid endometrial cancer.

**Supplementary Table 12. Demographic and clinicopathological characteristics of Zurich/Basel series according to *POLE* proofreading mutation**

|  | **Zurich / Basel**  (N=229) | | | |  |
| --- | --- | --- | --- | --- | --- |
| **Demographic / clinicopathological characteristic** | ***POLE* wild-type**  N=223 (97.4%) | | ***POLE* mutant***  N=6 (2.6%) | |  |
|  | N | % | N | % | *P^†^* |
| **Age, years** |  |  |  |  |  |
| Median (range) | 66 (33-88) | | 57 (52-89) | | 0.42 |
| **Tumor type** |  |  |  |  |  |
| EEC | 215 | 96.4% | 6 | 100.0% | 1.0 |
| NEEC | 8 | 3.6% | 0 | 0.0% |  |
| **FIGO stage** |  |  |  |  |  |
| I | 143 | 64.1% | 5 | 83.3% | 0.17 |
| II | 31 | 13.9% | 0 | 0.0% |  |
| III | 42 | 18.8% | 0 | 0.0% |  |
| IV | 7 | 3.1% | 1 | 16.7% |  |
| **Grade** |  |  |  |  |  |
| 1 | 147 | 65.9% | 2 | 33.3% | 0.004 |
| 2 | 48 | 21.5% | 0 | 0.0% |  |
| 3 | 28 | 12.6% | 4 | 66.7% |  |

**POLE* proofreading exonuclease domain mutations detected from sequencing exons 9 and 13 only (contain ~90% pathogenic variants).

*^†^ P* values represent comparison of *POLE* wild-type and *POLE* proofreading mutant groups calculated by unpaired t-test (age), Fisher’s exact test (tumor type), or χ^2^ test (other). EEC – endometrioid endometrial cancer, NEEC – non-endometrioid endometrial cancer. All statistical tests were two-sided.

**Supplementary Table 13. Patient outcome according to *POLE* proofreading mutation in Leuven series**

| **Outcome** |  | **Univariable analysis** | | **Multivariable analysis** | |
| --- | --- | --- | --- | --- | --- |
|  | **Events/total (%)** | **Hazard ratio**  **(95% CI)** | ***P**** | **Hazard ratio**  **(95% CI)** | ***P**** |
| **Recurrence** | |  |  |  |  |
| *POLE* wild type | 51/161 (31.6) | 0.14  (0.008 -2.34) | 0.17 | 0.18  (0.01 -3.11) | 0.24 |
| *POLE* mutant | 0/9 (0.0) |  |  |  |  |
| **Cancer specific survival** | |  |  |  |  |
| *POLE* wild type | 30/161 (18.6) | 0.24  (0.01 – 3.10) | 0.20 | 0.66  (0.04 - 11.39) | 0.78 |
| *POLE* mutant | 0/9 (0.0) |  |  |  |  |
| **Overall survival** | |  |  |  |  |
| *POLE* wild type | 36/161 (22.4) | 0.21  (0.01 – 3.60) | 0.28 | 0.53  (0.03 – 8.96) | 0.66 |
| *POLE* mutant | 0/9 (0.0) |  |  |  |  |

* Calculated using Cox proportional hazards two-sided test. Cox models use all informative cases irrespective of histology. Corresponding results from multivariable analyses following omission of *POLE*-wild-type NEECs from models are: RFS – HR=0.18, 95%CI 0.01-3.25, *P*=0.25; CSS – HR=1.07, 95%CI 0.52-21.95, *P*=0.97 (multivariable analysis of OS not done for EEC subset)

**Supplementary Table 14. Patient outcome according to *POLE* proofreading mutation in the TCGA series**

| **Outcome** |  | **Univariable analysis** | | **Multivariable analysis** | |
| --- | --- | --- | --- | --- | --- |
|  | **Events/total (%)** | **Hazard ratio**  **(95% CI)** | ***P**** | **Hazard ratio**  **(95% CI)** | ***P**** |
| **Recurrence** | |  |  |  |  |
| *POLE* wild type | 40/211 (19.0) | 0.164  (0.0083 -2.35) | 0.20 | 0.12  (0.0070 - 2.11) | 0.15 |
| *POLE* mutant | 0/18 (0.0) |  |  |  |  |
| **Cancer specific survival** | |  |  |  |  |
| *POLE* wild type | NR | NR | NR | NR | NR |
| *POLE* mutant | NR |  |  |  |  |
| **Overall survival** | |  |  |  |  |
| *POLE* wild type | 21/211 (9.9) | 0.29  (0.016 – 5.15) | 0.43 | 0.34  (0.018 – 6.48) | 0.48 |
| *POLE* mutant | 0/18 (0.0) |  |  |  |  |

* Calculated using Cox proportional hazards two-sided test. Cox models use all informative cases irrespective of histology. Corresponding results from multivariable analyses following omission of *POLE*-wild-type NEECs from models are: RFS – HR=0.18, 95%CI 0.01-3.08, *P*=0.24 (multivariable analysis of OS not done for EEC subset). NR – not reported

**Supplementary Table 15. Patient outcome according to *POLE* proofreading mutation in the Zurich/Basel series**

| **Outcome** |  | **Univariable analysis** | | **Multivariable analysis** | |
| --- | --- | --- | --- | --- | --- |
|  | **Events/total (%)** | **Hazard ratio**  **(95% CI)** | ***P**** | **Hazard ratio**  **(95% CI)** | ***P**** |
| **Recurrence** | |  |  |  |  |
| *POLE* wild type | NR | NA | NA | NA | NA |
| *POLE* mutant | NR |  |  |  |  |
| **Cancer specific survival** | |  |  |  |  |
| *POLE* wild type | 24/223 (10.8) | 0.73  (0.04 – 12.70) | 0.82 | 0.21  (0.01 – 4.26) | 0.31 |
| *POLE* mutant | 0/6 (0.0) |  |  |  |  |
| **Overall survival** | |  |  |  |  |
| *POLE* wild type | 57/223 (25.6) | 0.87  (0.17 – 4.48) | 0.86 | 0.42  (0.08 – 2.27) | 0.31 |
| *POLE* mutant | 0/16 (0.0) |  |  |  |  |

* Calculated using Cox proportional hazards two-sided test with Firth correction. Cox models use all informative cases irrespective of histology. Corresponding results from multivariable analyses following omission of *POLE*-wild-type NEECs from models are: CSS – HR=0.23, 95%CI 0.01-4.63, *P*=0.34. NR – not reported. NA – not available

**Supplementary Table 16. Comparison of *POLE* proofreading-mutant endometrioid endometrial cancers by grade in PORTEC studies and additional series**

|  | ***POLE* proofreading mutant* EECs**  (N=77) | | | |  |
| --- | --- | --- | --- | --- | --- |
| **Clinicopathological characteristic** | **Grade 1/2**  N=48 | | **Grade 3**  N=29 | |  |
|  | N | % | N | % | *P^†^* |
| **FIGO stage** |  |  |  |  |  |
| I | 44 | 91.7% | 22 | 75.9% | 0.14 |
| II | 2 | 4.2% | 2 | 6.9% |  |
| III | 1 | 2.1% | 3 | 10.3% |  |
| IV | 1 | 2.1% | 2 | 10.3% |  |
| **Depth of myometrial invasion** | |  |  |  |  |
| <50% | 17 | 35.4% | 15 | 51.8% | 0.15 |
| >50% | 28 | 58.3% | 12 | 41.4%^‡^ |  |
| Not known | 3 | 6.3% | 2 | 6.8% |  |
| **LVSI** | |  |  |  |  |
| No | 42 | 87.4% | 18 | 62.1% | 0.06 |
| Yes | 3 | 6.3% | 6 | 20.7% |  |
| Not known | 3 | 6.3% | 5 | 17.2% |  |
| **Recurrence** |  |  |  |  |  |
| No | 45 | 93.8% | 29 | 100% | – |
| Yes | 3 | 6.3% | 0 | 0% |  |

* *POLE* proofreading exonuclease domain mutations detected from sequencing exons 9 and 13 only in PORTEC, Leuven and Zurich/Basel series (contain ~90% pathogenic variants), and whole exome sequencing in TCGA series.

*^†^ P* values represent comparison of informative grade 3 and grade 1/2 ECs by or χ^2^ test (stage) and Fisher’s exact test (invasion, LVSI). All statistical tests were two-sided.

^‡^ May underestimate the true frequency of deep myometrial invasion in high-grade *POLE* mutant tumors due to exclusion of grade 3 ECs with >50% myometrial invasion from PORTEC studies. In *POLE*-mutant ECs from the unselected additional series deep myometrial invasion was more common in grade 3 than in grade 1/2 tumors (75% vs. 25%, *P*=0.04, Fisher’s exact test).

LVSI – lymphovascular space invasion.

**Supplementary Figure 1. Outcome according to *POLE* proofreading mutation in Leuven series**

Probability of differential recurrence-free survival (A) and cancer-specific survival (B) according to *POLE* proofreading mutation status are shown. *P* values are obtained by log-rank test. All statistical tests were two-sided.

**Supplementary Figure 2. Recurrence-free survival according to *POLE* proofreading mutation in TCGA series.**

Two-sided *P* value obtained by log-rank test

**Supplementary Figure 3. Cancer-specific survival according to *POLE* proofreading mutation in Zurich/Basel series.** Two-sided *P* value obtained by log-rank test.
